# Collaboration Routines and Workflows in a National Electronic Prescription System: Qualitative Study

**DOI:** 10.2196/56558

**Published:** 2025-06-10

**Authors:** Kristine Lundhaug, Arild Faxvaag, Randi Stokke, Hege Kristin Andreassen

**Affiliations:** 1 Department of Health Sciences in Gjøvik, Centre for Care Research East Faculty of Medicine and Health Sciences Norwegian University of Science and Technology (NTNU) Gjøvik Norway; 2 Department of movement science and neuromedicine Faculty of Medicine and Health Sciences Norwegian University of Science and Technology (NTNU) Trondheim Norway; 3 Institute of Health and Care Sciences Faculty of Health Sciences The Arctic University of Norway (UiT) Tromsø Norway

**Keywords:** e-prescription, sociotechnical system, ethnographic study, health professionals, delegation, enabler, workarounds, mediator

## Abstract

**Background:**

Electronic prescription (EP) systems are supported by health authorities worldwide, and most European countries have started implementing or have fully implemented such systems. However, few studies have explored how EP systems affect the work of health care professionals (HCPs) across institutions and levels in health and care services.

**Objective:**

This study aims to explore changes in HCPs’ roles, tasks, and responsibilities related to medication management by following a national EP system through different contexts and levels in health and care services.

**Methods:**

Through a qualitative study with an ethnographic approach, including participant observations and individual interviews, this study followed an EP technology in an intermediate unit, an emergency unit at a hospital, and a municipal home care service in Norway. Participant observations were conducted for 6 weeks in the intermediate unit, the emergency unit at the hospital, and the home care service. During the observations, 20 individual interviews with HCPs were conducted. For the analysis, we leaned on a stepwise-deductive inductive approach, using the concepts of *delegation* and *enabler* as theoretical tools to explore how roles, tasks, and responsibilities were being distributed within the sociotechnical system of which EP formed part.

**Results:**

The results showed that physicians were overall satisfied with the Norwegian EP system and expressed satisfaction that some of their previous tasks were now delegated to the EP system, allowing a more efficient workflow on their side. In contrast, the home care service managing medication on their patients’ behalf described several challenges and reported an increased workload related to medication management. Home care nurses often became mediators between the general practitioners and the pharmacies to ensure patient safety. The home care nurses also developed EP-based work-arounds to enable the Norwegian EP system to work.

**Conclusions:**

This study revealed that Norwegian the EP systems altered daily medication management routines, removing tasks from physicians and creating and delegating new roles, tasks, and responsibilities to home care nurses. Drawing on theoretical concepts (delegation and enabler), this study offers insights into the changing distribution of roles, tasks, and responsibilities following in the wake of implementing national EP systems.

## Introduction

### Background

The increasing focus on and development of digital technologies is visible and present in various facets of our lives, and health care services are no exception. The health care sector has experienced different technological innovations and has implemented technological tools that are built to ease, help, or solve problems in the everyday workflow of health care professionals (HCPs). The Norwegian national electronic prescription (EP) system is a technological tool that aims to improve medication-related workflows and increase patient safety. The Norwegian EP system is a national eHealth system for securely transferring EP information that “supports the electronic flow of information related to prescribed medications” [1]. It contains all the necessary information about the prescribed medication, including the medication’s name, dosage instructions, quantity, and the prescriber’s details. EP system initiatives are also driven by concerns about regulating prescriptions for cost-control reasons [1]. EPs have replaced traditional paper prescriptions. Instead of physically handing over paper-based prescriptions to patients, HCPs with prescriptive authority generate and transmit prescriptions electronically, thereby digitalizing collaboration routines and workflows related to medication management.

EP systems are prioritized and supported by health authorities around the world [1]. The Nordic countries are “considered to be leaders in Europe in the field of ePrescribing” because they have had an “adoption rate of 80% or higher” in 2013 [2]. In primary care facilities in Sweden and Denmark, nearly all prescriptions are digital [3,4]. A study presenting the state of national EP systems in 19 European countries found that most EP systems in different countries had 1 national EP project, with a similar architecture. However, the EP systems varied in authentication procedures and advanced functions, such as integration with a national electronic health record (EHR) [5]. The stages of maturity of EP systems vary across European countries, ranging from early adoption to routine use [1]. The national EP systems across different countries with different health systems vary in actors, functionality, and access due to national regulations. “Variations relate to: what constitutes prescriptions drugs, who can issue a prescription, what is the minimum required content of a prescription, who can dispense a prescription, how medications are reimbursed” [1].

**Figure 1:**
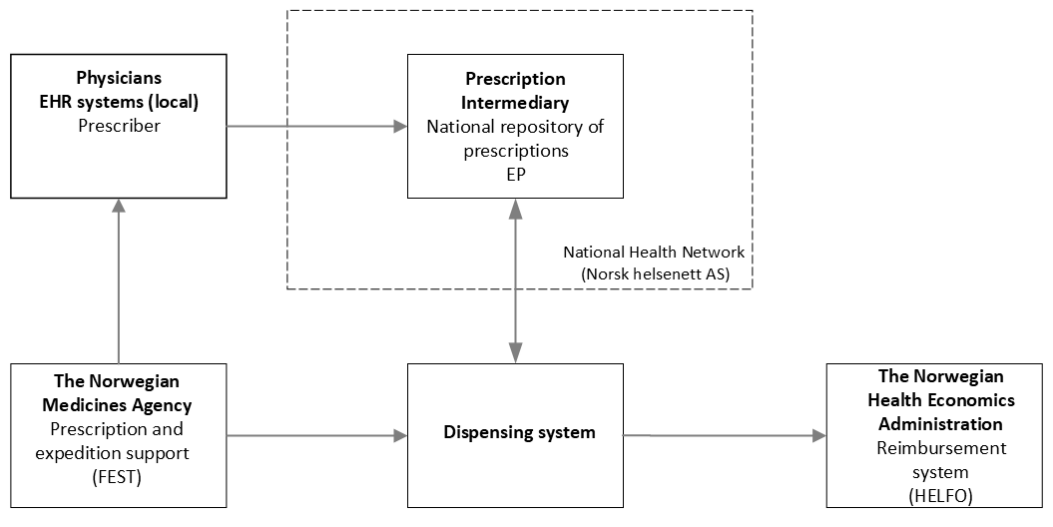
The workflow in the Norwegian electronic prescription (EP) system. EHR: electronic health record.

In Norway, where this study was conducted, the EP system was implemented in primary and specialist health care by 2015 [1]. As of 2023, approximately 93% of the prescriptions are EPs used by general practitioners (GPs), hospitals, pharmacies, and bandagists [6]. In Norway, the workflow for EPs in the EP system is that the prescription is generated through the local EHR systems. The EP is transmitted to a central Prescription Intermediary database, a national repository of prescriptions. The Prescription Intermediary database is used by the prescriber and dispensing systems (pharmacies) to check whether the prescription is valid and can be dispensed. The Norwegian Medicines Agency provides prescription and expedition support, and the Norwegian Health Economics Administration provides a reimbursement system, as illustrated in Figure 1. The EP system interacts with the national Summary Care Record and different local EHRs used in municipalities and specialist health care. To be valid, prescriptions must comply with the regulations [7]. According to the regulations, a prescription is valid for 1 year from the date it was issued, except for hormonal contraceptives (valid for 3 years) and prescriptions for antibiotics (valid for 10 days) [7].

The advantages and goals of EP systems, such as efficiency, accuracy, accessibility, and safety, are often highlighted because EPs reduce the risk of errors in ordering and dispensing medicines and increase patient safety through safer medication use by reducing prescription errors. Previous research has shown that EPs can improve physicians’ workflow, reduce medication errors, and increase pharmacy efficiency [8-12]. However, a study conducted in Norway after the implementation of the EP system found that “pharmacists at Norwegian pharmacies yearly prevent over 400,000 prescription errors of potential clinical importance” [11]. Studies have also shown that physicians, GPs, and pharmacists are generally satisfied with the use of EP systems in their work [13,14]. A literature review [15] revealed several benefits of EP systems, such as reducing medication errors, improving the quality of health care services and patient safety, and improving the workflow of prescribers. However, the literature review also showed that if the EP system is not adequately implemented, it can reduce workflow efficiency and threaten patient safety. Researchers have provided insights into how using the EP system has led to work-arounds for HCPs [16,17]. One study found that HCPs in a hospital setting design work-arounds that involve paper and other technologies (Microsoft Word) for intermediary information storage to bridge information gaps in their EP system [16]. A study in ambulatory care practice found that due to functional limitations of the EP system, nurses created an EP-based work-around for the necessary work, including calling the pharmacy directly [17]. A large-scale survey of HCPs (primary care physicians, specialized physicians, and pharmacists) regarding perceptions of EP system showed that, collectively, they perceived the EP system as having a positive impact on the overall prescribing process because, compared to handwritten prescriptions, the EP system simplified procedures and reduced errors [18]. The literature review mentioned earlier [15] and other previous studies and reviews [16,17,19,20] show that, to date, most studies on EP systems have been conducted in separate practice settings, such as ambulatory care, hospital-based care, community care settings, and pharmacies.

To our knowledge, few studies on EP systems have included a variety of HCPs across health and care settings within a single study. However, several studies have been conducted on information exchange across the health and care services and their impact on health care delivery [21-23]. A nationwide quantitative study on nurses’ experiences transitioning older patients from hospital to community care found that community nurses were less satisfied with the information exchange on patients’ conditions and needs than hospital nurses [21]. Studies on systems that are considered helpful and effective found that electronic information exchange is not always enough in the discharge planning of patients between hospital and municipal health care. Hence, using the telephone was a necessary supplement for providing accurate and nuanced information about the patient [24]. Studies have also found that electronic systems do not sufficiently support cross-sectoral communication, and that patient information may be prevented from being shared because the systems are not integrated [23]. Communication and collaboration between nurses and physicians are essential in health care delivery, but there are challenges associated with them [25]. Different factors affect collaboration, such as respect and trust, the lack of understanding about the roles and tasks of each other’s professions, and the unequal power between them [26]. “Nurses were often viewed as ‘handmaidens’ of physicians, while physicians were perceived as leaders of the healthcare team” [26].

The increasing use of eHealth and information systems has an impact on HCPs’ work. However, there is still little knowledge about how the roles of HCPs change among them as a potential effect of eHealth and information systems [27]. To fully understand how digital systems, such as an EP system (meant to be used across health and care service), are played out in contemporary health care, we must explore the system across practice settings, institutions, and disciplines. This study follows EP technology by exploring the roles, tasks, and responsibilities of the HCPs involved in 3 different contexts of health care: an intermediate unit, an emergency unit at a hospital, and a home care service. The research question examined is as follows: How does the national EP system play out across HCPs and health and care services in everyday work?

### Theoretical Concepts: Delegation and Enabler

Theoretically, this study draws on concepts from science and technology studies, an interdisciplinary academic field that studies the relationship between science, technology, and society, which entails that the social and the technological aspects are intertwined [28,29].

EP systems are complex systems integrated with other technological systems that involve several human actors across the health care sector. Hence, science and technology studies are an interesting point of departure for the academic analysis of these technologies. In our analytical work, 2 concepts prove particularly useful to explore changes in cross-institutional workflows: *delegation*, as used by Latour [30], and *the nurse as* *enabler*, from *The Invisible Work of Nurses: Hospitals, Organisation and Healthcare* by Allen [31], which draws on a practice theory approach. These theoretical tools helped us unpack and highlight interesting nuances in the workflows and collaboration routines in the sociotechnical system of EP.

The concept of delegation by Latour [30] can provide insights into a sociotechnical system where tasks, roles, and responsibilities are transferred and distributed between technology and humans. Delegation can occur both among human actors and between human and nonhuman actors; this entails that it is not only humans who can affect technology but technology can also affect and shape human actions. Delegation is not a one-time event, but a constantly ongoing process within social networks through interactions between technologies and humans. The concept of delegation is particularly useful when exploring digitalization. This can involve the transfer of power, decision-making, and responsibility from one entity to another.

The concept of nurses as enablers by Allen [31] points to nurses’ vital roles in facilitating medical treatment and care through their constant coordination work. Allen [31] highlights the multifaceted roles of nurses in health care. According to Allen [31], nurses play an essential role in coordination and collaboration around patients, and patient safety is a fundamental aspect of the concept of nurses as enablers. Furthermore, nurses enable effective collaboration among HCPs, ensuring the continuity of care across various health care settings. Allen [31] argues “...that nurses are the network builders in healthcare systems. Barely anything happens which does not pass through the hands of a nurse.”

This study aims to follow the Norwegian EP system across various levels, contexts, and professions by applying 2 different concepts, delegation and enabler, to explore the role, tasks, and responsibilities of the HCPs involved in the 3 different contexts: an intermediate unit, an emergency unit at a hospital, and the home care service.

## Methods

This study had a qualitative design with an ethnographic approach, which included participant observations and interviews. The ethnographic approach was useful when following EP technology in different contexts by contributing contextual insights and providing a more profound understanding by combining observations and interviews when engaging with HCPs in their practice.

### Study Context and Settings

The Norwegian health care system is divided into specialist health care and primary health care [32]. The hospital sector is run by the national health authorities and is responsible for the specialist health care service. Primary health care is provided under the responsibility of the municipalities, and they have responsibility for, among other things, nursing homes, home care services, intermediate units, and GPs [32]. This study was conducted in an intermediate unit, an emergency unit at a hospital, and a home care service in Norway from February to September 2021, with 1 follow-up interview in February 2023. All the units were located in the same municipality.

Norway currently has 2 national eHealth systems, the Summary Care Record and the EP system, but the Summary Care Record have not been implemented in all the municipalities. The municipality in this study was strategically chosen because it had implemented both national eHealth systems. Purposeful sampling was used to recruit the different units to select information-rich cases and participants strategically relevant to the research questions [33]. The *intermediate unit* was purposefully chosen because the unit serves as a bridge in the continuum of care across different health care service levels, particularly supporting the transition from hospital to home. Its primary functions are to prevent unnecessary admissions to the hospital, shorten hospital stays, facilitate timely discharges, create individualized care plans, and promote independent living [34]. The HCPs working in the intermediate unit were nurses, physicians, physiotherapists, and occupational therapists. The typical patient stay is 2-3 weeks [34]. The intermediate unit also offered municipal inpatient acute care for patients requiring urgent assistance or observation for up to 72 hours. The *emergency unit* at the hospital was purposefully chosen as the unit provided urgent care to patients needing immediate medical attention. Physicians from various specialties and nurses staffed it. Patients are typically referred by their GPs, the Emergency Medical Communications Center, or the emergency room. Patients may also arrive at the unit independently, by ambulance, or by ambulance helicopter. The emergency unit operates 24×7 to ensure continuous care. The *home care service* was purposefully chosen because it offered a range of health care and assistance to individuals living at home, helping them with daily activities. These services may include medication management, medical procedures, personal care, mobility assistance, and meal support. The home care service aims to enable individuals to remain in their homes, even when they require help with specific tasks. Nurses and professional caregivers primarily provide the care.

The units were purposefully sampled to get insights across the health and care service, and some units were chosen because they had a high patient flow in and out of the units that may require a frequent gathering of patient information and therefore may increase the use of the national eHealth systems.

Participant observations were conducted for 2 weeks in the intermediate unit from February 2021 to March 2021. During the participant observations, individual interviews with 5 nurses and 4 physicians were conducted. Data collection in the emergency unit at the hospital was conducted from August 2021 to September 2021, with 3 weeks of participant observation, during which 5 individual interviews with physicians and 2 individual interviews with nurses were conducted. In September 2021, 1 week of participant observation was conducted at the home care service, during which 4 individual interviews with nurses were conducted. Moreover, 1 follow-up individual interview was conducted in February 2023 with one of the nurses previously interviewed at the home care service. The necessity for a follow-up interview arose during the analysis phase when there was a need to ask some follow-up questions concerning medication management in the home care service.

### Content of Observational Studies and Individual Interviews

This study used participant observations to explore how a national eHealth system was used and how patient information was shared between and across municipal and specialist health services. Observational studies are useful for gaining insights into different aspects of the workplace [35] and understanding the context being studied [33]. Observing the settings, activities, and HCPs in their everyday work can provide insights into a workflow that is not well known. Participant observations entailed that the first author followed the HCPs around the units and in the medication room and participated in daily and weekly meetings. However, the participant observation mainly took place in the HCPs’ workspaces for computer work. The workspace consisted of a desk with a computer that the HCPs used to gather and document patient information, except for the home care service, which also used a mobile unit linked to the EHR. Home care services used a mobile unit before, during, and after home visits. On the mobile unit, they could see the patients’ addresses and EHRs and document patient information after a home visit. During the participant observation, the first author was conscious of where the observation occurred and strived not to stand in the way of the HCPs’ workflow but simultaneously had to be able to see the computer screens. Before the participant observation started, the HCPs working in the different units were informed about the study, the background, and the role of the first author. During the observation, informal conversations occurred where the HCPs wanted to show the first author something on the computer, for example, during the medication reconciliation, when a physician showed the different sources they used and how the sources sometimes had a discrepancy between them.

The first author took field notes during the participant observations in a small pocket-sized notebook but did not write the field notes in front of the HCPs. This was a conscious decision to avoid disturbing or making the HCPs aware of the first author’s reaction to a situation. The field notes were often written when the HCPs left the workspace or discreetly in suitable situations. The field notes were written throughout the day, and at the end of each day, they were rewritten as comprehensive field notes. The field notes consisted of what activity the HCPs were engaging in, what the participants were doing and saying to themselves or others while working on the computer, who talked to whom under different circumstances, and what kind of technology was used (when, how, and in which setting).

Individual interviews were conducted as focused interviews. According to Tjora [35], focused interviews can be “suitable for work-related studies”; the interviews took place during participants’ work hours. Tjora [35] pointed out 3 criteria that can be useful in assessing whether a focused interview is more suitable than a lengthy, in-depth interview. The three criteria were as follows: (1) if the topic is very limited, (2) if trust can be gained relatively early in the interview, and (3) if the topics are not very sensitive or difficult. Because the focused interviews took place during the participant observations, the HCPs were already familiar with the study. A couple of days into the participant observation, the first author asked HCPs on duty if they wanted to be interviewed, either on the current shift or another shift, while the participant observation was taking place. The units were, as mentioned, purposefully sampled, but this also applied to the participants. The participants were purposefully chosen because they worked in the units, and the first author asked both physicians and nurses from the emergency and intermediate unit if they wanted to be interviewed. The reason for including different professions was to get different perspectives through various professions in various contexts across the health and care service. This increased the chances that the HCPs had different experiences with the national eHealth systems. The work experience of the nurses ranged from 8 months to 20 years, and the work experience of the physicians ranged from 2 months to 18 years. In the home care service, the employees consisted of both nurses and professional caregivers. The nurses in the home care service were the only HCPs invited to participate in interviews, as they were the only ones working in the medication room where they managed patients’ medications. The focused interviews were all conducted in Norwegian at the participants’ workplace during work hours, and the location was either a meeting room or an office.

The research team consisted of all 4 authors, who were all university academics with backgrounds in sociology, medicine and health informatics, and nursing. The research team had regular meetings before, during, and after the data collection. These meetings made it possible for the research team to express different opinions, have discussions, raise questions, and challenge preconceptions, and these meetings were particularly useful because the research team members had diverse backgrounds. The research team developed the interview guide; although we did not pretest the interview guide, the questions were thoroughly discussed with the research team, and we strived for the interview guide to be semistructured, with open questions. During the participant observations, there could have been situations that occurred that the first author wanted to ask questions about during the interviews, either for clarification or to get a deeper understanding of the situation. The interviews started with warm-up questions, such as “How long have you worked as a health care professional?” and “What is your position here?” Subsequently, questions such as “How familiar are you with e-prescription?” and follow-up questions about the type of experience HCPs had with EPs were asked. The interviews ranged from 17 to 80 minutes, with most interviews lasting for an average of 30 minutes. All interviews were audio recorded and transcribed verbatim by the first author. The transcripts and field notes were written in Norwegian to maintain proximity to the data during analysis. For this paper, the research team translated the quotes into English. The research team strived to retain the meaning of the quotes during the translation process by going back and forth between the Norwegian and the translated quotes in English. This was a challenging process at times because the participants sometimes used terms and expressions that were difficult to translate.

### Analysis

The analysis process was inspired by a stepwise-deductive induction (SDI), which was used to analyze the field notes and transcripts [35]. The field notes and transcripts were imported into NVivo software (Lumivero), which organized the data material for analysis. The SDI analysis began with an inductive interpretation and gradually incorporated a theoretical perspective throughout the analytical phase. The first step of the analysis was to create codes generated from empirical data rather than from theories, hypotheses, or themes from the interview guide [35]. The first author coded the interviews and field notes into detailed inductive coding close to the participants’ statements to reflect the meaning or descriptions of concrete situations, which resulted in approximately 700 empirical codes. The second step involved grouping the codes with thematic meanings, resulting in 9 code groups. On the basis of these code groups, the main code group relevant to this paper was *EP—inhibits and promotes workflow*, which was particularly rich in data and also interesting considering the research question. Thus, this code group was suggested for a more in-depth theoretical analysis. Subsequently, we departed from inductive-inspired close coding and approached the data from a more deductive angle. All the authors participated in joint analytical workshops, contributing to deconstructing and building analytical constructs for the observations and quotes within this particular code group. The codes in the code group were examined inductively, and we incorporated different theoretical concepts and previous research. The 2 theoretical concepts, delegation and the nurse as an enabler, were applied in the last step (after the abductive phase) to help focus on the analytical gaze, structure the analysis, and group the findings into 2 themes: *delegation to make the workflow efficient* and *enabling the technology.* The 2 themes and examples of some of the associated codes are presented in Textbox 1. Furthermore, the 2 theoretical concepts were used as conceptual lenses in discussing the results.

**Examples of analytical work (themes and corresponding codes).**

**Delegation to make the workflow efficient**

- A revolution
- Surprisingly little problems
- If it is not down, it works
- It is efficient
- It works as long as they are renewed

**Enabling the technology**

- My job is to remind the doctor
- If urgent, we call
- Prescription renewals—doctors’ responsibility
- Lot of communication with general practitioners
- I do not understand these general practitioners
- We have the role of mediators

### Ethical Considerations

The Regional Committee for Medical and Health Research Ethics approved the exemption from the duty of confidentiality (reference number 141144), and the Norwegian Center for Research Data (reference number 919576) approved the study before we began the data collection process. The municipality’s health and welfare leader approved the participation in the research project. Following this approval, communication between the first author and various units was initiated. The units were notified of the study and subsequently given the option to participate. The head of the emergency medical care department of the hospital was contacted, informed about the study, and agreed to participate. The hospital’s data protection supervisor registered the study to ensure compliance with the data protection regulations. Before participating in the study, all participants were provided with written and oral information about the research and were notified about their right to withdraw without providing a reason. Written informed consent to participate was obtained from all the participants. Moreover, they were assured of the confidentiality of the transcribed data (anonymized systematically) and publications from the study. The participants were not offered compensation for participating in this study. The first author communicated the research project details to all participants, and they all agreed to the individual interviews being audio recorded.

## Results

### Overview

A total of 6 weeks of participant observation and 20 individual interviews were conducted with HCPs; Table 1 provides the characteristics of the participants. In this paper, the term HCP is a collective term that includes both nurses and physicians; otherwise, terms such as nurse or physician will be explicitly stated.

In our analysis, we focused on following the technology in different contexts across health and care service levels to explore HCPs’ roles, tasks, and responsibilities within the sociotechnical EP system. In the subsequent sections, the 2 themes, *delegation to make the workflow efficient* and *enabling the technology*, identified through the SDI analysis, are presented with empirical examples interpreted through the concepts of delegation and the nurse as an enabler.

**Table 1:** Study participants’ characteristics.

Health professional	Workplace	Work experience
Physician	Intermediate unit	2 mo
Physician	Intermediate unit	8 mo
Physician	Intermediate unit	9 y
Physician	Intermediate unit	18 y
Nurse	Intermediate unit	6 y
Nurse	Intermediate unit	3.5 y
Nurse	Intermediate unit	8 mo
Nurse	Intermediate unit	12 y
Nurse	Intermediate unit	10 y
Nurse	Home care service	2 y
Nurse	Home care service	17 y
Nurse	Home care service	18 y
Nurse	Home care service	3 y
Physician	Emergency unit (hospital)	2 y
Physician	Emergency unit (hospital)	1.5 y
Physician	Emergency unit (hospital)	1 y
Physician	Emergency unit (hospital)	1 y
Physician	Emergency unit (hospital)	9 y
Nurse	Emergency unit (hospital)	20 y
Nurse	Emergency unit (hospital)	11 y

### Delegation to Make the Workflow Efficient

Physicians in the intermediate and emergency units were satisfied with the EP system and were found to function well in everyday work. A physician reflected on how “easy” the EP was compared to the paper prescription:

It has worked very well; getting it was a little revolution. I have written many prescriptions by hand, so this is brilliant. It has been surprisingly well-functioning and has had few technical problems.

Physician; intermediate unit

The physicians’ only problem with the EP was when the system was “down.” An EP is typically created when a physician sends the prescription electronically to a central database called a Prescription Intermediary. When the physician spoke about the system being “down,” they meant that their local EHR integrated with the Prescription Intermediary was sometimes unstable and lost contact, so the physicians were unable to send the prescriptions electronically. When the system was “down,” and the physicians could not prescribe EPs, they could still write paper prescriptions or send an electronic message to the GPs. One of the physicians expressed the following:

I have solved this by writing a note of apology to the GP along with the discharge summary. I say that I am not able to write an e-prescription because the system is down. And I ask, can you arrange prescriptions for the patient?

Physician; intermediate unit

The nurses had different experiences with EP depending on their context. In the emergency unit, the nurses considered anything related to prescribing medication to be a physician’s task and were not involved. The nurses in the intermediate unit had a different attitude toward physicians’ roles regarding EPs. Nurses stated that they reminded physicians to write an EP before the patient was discharged:

The only thing I use e-prescriptions for as a nurse is to remind the doctor that they must remember to write an e-prescription before the patient is discharged. This is to make sure the patient gets it. It is the doctor’s responsibility, but it is my job to remind the doctor to write the e-prescriptions.

Nurse; intermediate unit

When a patient was discharged from the emergency or intermediate unit and transferred to a home care service, the home care service often managed the patient’s medications. The home care service received an overview of the patient’s medication in the medication list, which was part of the discharge summary from the emergency or intermediate unit. When physicians added new medications to a patient’s medication list, they created EPs for the new prescriptions. A discharge summary was also sent to the GP, giving the GP the same information as the home care service. To obtain an overview of the patient’s medications, home care nurses started with a home visit, where they talked to the patient and sometimes to the next of kin and collected the medicines the patient had at home. The patient signed a consent form for the home care service to manage their medication. Home care nurses also relied on the medication list, which was part of the discharge summary, to obtain an overview of medications. When home care nurses had an overview of the patient’s medication list, they used an online pharmacy platform to order medications.

The home care nurses ordered the medication through the online pharmacy platform, and the platform was integrated with the Prescription Intermediary to verify whether the EPs were valid. When the EPs were valid, the home care nurse could continue to order the medication, and the pharmacy could deliver it to the home care service.

### Enabling the Technology

**Figure 2:**
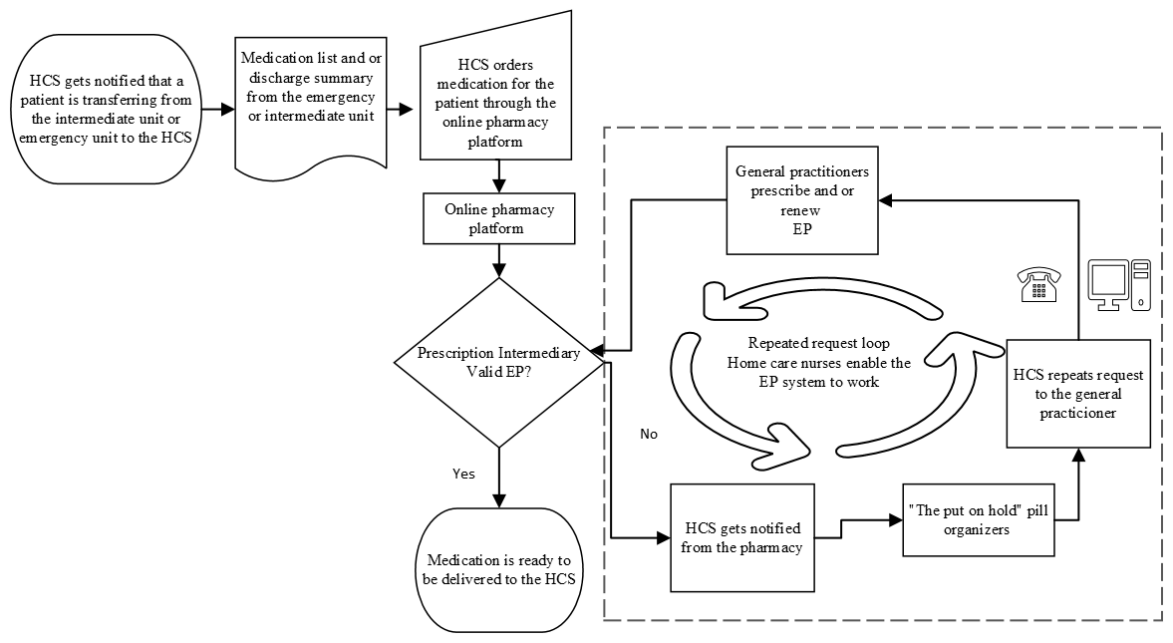
A flowchart of the medication management in the home care service (HCS) showing how the electronic prescription (EP) system delegates tasks, roles, and responsibilities to the home care nurses and how the home care nurses enable the technology.

When an EP was no longer valid, which often occurred, the pharmacy notified the home care nurses through the online pharmacy platform when they tried to order an invalid EP. The home care nurse then had to contact the GP through electronic messages or phone to remind the GP to write a valid EP so that home care nurses could order the medications and administer them to patients, as illustrated in Figure 2.

Home care nurses contacted the GPs every time there was an invalid EP:

I’m doing three things simultaneously: on the phone with the doctor and the pharmacy and doing other things...I can sit for over an hour with one patient’s medications due to changes on the medication list.

Home care nurse

The home care nurses’ experiences with EPs that were no longer valid were not an isolated problem for patients discharged from the emergency and intermediate units. They also experienced this when the GP changed the patient’s medication list or when patients were transferred from other hospital units. A home care nurse stated that they could receive an electronic message from the GP about adding some medications to the patient medication list, but the prescription was not valid:

I don’t understand these doctors. You can get an electronic message that says that this patient is starting on these medications. Then we contact the pharmacy, but the EP is invalid or not a prescription at all.

Home care nurse

Home care nurses spent a lot of time repeatedly contacting GPs about invalid EPs, which the nurses found time consuming and unnecessary, but important at the same time. One of the nurses explained the following:

Medication management is one of the most important tasks in our job.

Home care nurse

Nurses in the home care service reported that they were mediators in medication communication between the pharmacy and the GP. One nurse explained the following:

We have become a mediator. We must repeatedly remind the GPs when a patient has been to the hospital and there have been medication changes. We become a mediator in the communication. It should not be necessary because we receive the same information [discharge reports] as the GPs when a patient has been discharged.

Home care nurse

The home care nurses’ roles as mediators were also evident when a patient needed an EP renewal, as illustrated in Figure 2. When a patient’s prescription has expired and the GP must renew the EP, the pharmacy sends a message to the home care service. Home care nurses must then contact the GP to ensure that the medication is renewed by sending an electronic message or calling the GP to remind them:

The physician is always responsible for renewing prescriptions and notifying us what medication the patient is on. We must wait for a message from the doctor before we can do anything and follow the medication list when we dispense the medication into pill organizers. If we have not received the right medication list, or the medication is not renewed at the end of the workday, we write a note to the next nurse on duty to follow up. In these situations, important messages can be missed.

Home care nurse

The home care service had a medicine room where home care nurses managed the medication. The medicine room was a cramped room consisting of 2 computers, a medicine cabinet, and shelf units with baskets containing the names of patients and the medications they used. The nurses had created a system and dispensed the medication into weekly pill organizers with the patients’ names on them. When the EPs were valid, the pill organizer was placed in a specific place on the shelf, indicating that the pill organizer was ready for double checking. The pill organizer was double checked, that is, another nurse verified that the medication list and the weekly pill organizer were correct.

When the EP was invalid, home care nurses could not order the patients’ medications through the online pharmacy platform. During the observation period, it became apparent that the home care nurses had developed a separate routine for handling invalid EPs because they could not continue to dispense the medication into the weekly pill organizer. The pill organizer was then put in a specific, different place on the shelf, symbolizing that medication dispensing was not “done,” but “put on hold,” until the EP was valid. The “put on hold” pill organizer had yellow Post-it notes stuck on it with a handwritten message as follows: “This and this medication is missing, have sent an electronic message to the GP.” The pill organizers sat on that shelf until the EP was valid; then, the home care nurses could dispense the “missing” medication into the pill organizer and move it to the part of the shelf that symbolized that it was ready for double checking.

The dotted line box in Figure 2 illustrates the EP-based work-around that the home care nurse developed when managing medication on their patients’ behalf. The necessity of the “put on hold” pill organizer delayed the home care nurses’ medication management as they had to write notes concerning the medication on Post-it notes, stick them onto the patients’ pill organizers, and put them on the “put on hold” shelf. Following this, the home care nurses then had to spend time repeatedly contacting the GPs via calls or sending electronic messages. This “repeated request loop” had become part of the home care nurses’ work routine when the EP was invalid.

By following Norwegian EP technology in different contexts and across levels, the varying experiences of HCPs showed that, overall, physicians were satisfied with the EP system. Some of the physicians’ work tasks were delegated to the technology, and the technology delegated new tasks that appeared in other places in the sociotechnical system which became visible through the home care nurses medication management in the medication room. The EP system seemed to work well for HCPs until it came to the home care service that managed medication on behalf of their patients. At this point, problems began to occur within the EP system. New tasks were delegated to the home care service, where the home care nurses were delegated the role of mediators between the pharmacy and the GP when the EP was invalid. Nurses played a key role as mediators and enablers of the EP system. Home care nurses enabled the EP system to work in the complex, multidisciplinary, and multi-institutional health care system in Norway by taking on new tasks delegated to them in the workflow and creating new collaboration routines in medication management to ensure patient safety.

## Discussion

### Principal Findings

This study explored the sociotechnical EP system by following it across the health and care service levels through an ethnographic approach. Using the delegation concept proposed by Latour [30] sensitizes our analysis of how and what the EP system delegates in a network of human and nonhuman actors. “Delegation is particularly concerned with how tasks and responsibilities are distributed between people and technology” [36]; in this study, delegation is used to explore and understand how roles, tasks, and responsibilities have been delegated between HCPs and the EP system. The concept of the nurse as an enabler by Allen [31] helped us unpack the invisible work of home care nurses when dealing with medication management, revealing that the fully digitalized Norwegian EP system is perhaps not fully digitalized after all; there is still a lot of human coordination involved in making collaboration and cross-institutional workflows possible. The concepts of delegation and nurses as enablers were appropriate to highlight nuances in our findings and help us explain and understand how roles, responsibilities, and work tasks transfer between human and nonhuman actors in the EP system. Our findings provide an overarching story of how the Norwegian EP system plays out in various local contexts, with different consequences for different HCPs.

### Comparison With Prior Work

Because of the digitalization of part of the physicians’ work tasks from paper prescriptions to EPs, physicians have delegated part of the prescription work to the EP system, which involves delegating the responsibility for safely handling patient information to the technology. When following the EP technology in the intermediate and emergency units, all HCPs seemed satisfied with the EP system because it eased the workflow for the physicians. This shows that some human tasks, such as handwritten paper prescriptions, are delegated from humans to nonhuman technology, making the prescription electronic [30]. The findings show that the physicians were pleased with the EP system overall, which aligns with previous studies showing that physicians were satisfied with the EP system [14,37].

The concept of delegation [30] is useful when exploring the EP system technology. It contributes to understanding why and how the delegation of roles, tasks, and responsibilities unfolds across the health and care service and the consequences for different HCPs. When the technology was applied across health care service levels and to the home care service, the home care nurses were delegated new roles, tasks, and responsibilities through the EP system because the nurses managed the medication on the patient’s behalf. New roles, tasks, and responsibilities delegated to home care nurses played out during medication management. Home care nurses order medication through the online pharmacy platform, and often the EPs are invalid or have not been prescribed. Home care nurses often experience discrepancies among the medication list in their systems, the medication list following the discharge report, and invalid EPs. Kerstenetzky et al [38] found in their literature review that patients transitioning between health care settings often had medication discrepancies. Another study showed that, for patients transitioning between levels of care, the “medication lists were perceived as fragmented, complex, risky and time-consuming, as well as causing uncertainty” [39]. Another study found that to confirm or provide the correct medication list, pharmacists, nurses, and physicians had to depend on others before they could perform their work tasks, which involved work-arounds to obtain the information they needed to ensure medication safety [39]. This is supported by our finding that home care nurses function as mediators. A Norwegian study found that “88% of patients [had] at least one discrepancy in their medication list” [40] among the GP, home care service, and dispensing pharmacy. Home care nurses are delegated the responsibility of ensuring that patients receive medications, and our findings show that they take on this responsibility to ensure patient safety through medication management, one of the most important aspects of the job, according to home care nurses themselves. This illustrates how nurses play key roles in medication management. We found that when EP system technology plays out in everyday work, important sociotechnical tensions can arise in the cross-institutional and cross-professional collaboration routines and workflows in medication management.

A study on home care nurses describes their activities as “tie up loose ends and collect information” [41] and underlines how the home care nurses act as coordinators and intermediators among GPs, hospitals, and patients when it comes to finding the right medications [41]. This is in line with our findings and demonstrates that home care nurses are involved in complex roles, tasks, and responsibilities. Home care nurses found new ways for the EP system to work by taking on the role of enablers by mediating communication between the GPs and pharmacies if the EP was invalid. To enable the EP system to work, home care nurses developed EP-based work-arounds in the medicine room, when EPs were invalid, in anticipation of completing their job—which involved managing patients’ medications by dispensing them into pill organizers. When an EP was invalid, the home care nurses developed work-arounds, the “repeated request loop,” as illustrated in Figure 2. This process involved repeatedly contacting GPs regarding invalid EPs. Pill organizers with handwritten Post-it notes were placed on the “put on hold” shelf, indicating that the medications had not yet been dispensed due to an invalid EP. This work-around was a routine part of the home care nurses’ workflow when dealing with invalid EPs. Simultaneously, nurses function as mediators by reminding the GP to send a valid EP. This circular motion, as illustrated in Figure 2, is aligned with the description of nurses as obligatory passage points by Allen [31], as almost everything passes through a nurse’s hands. Thus, the home care nurses enable the EP system technology.

Our findings show that, for home care nurses to ensure medication safety, extra effort is required to enable the EP system to work, including work-arounds. Other studies have shown that EP-based workarounds often occur because of the system’s functional limitations and the lack of flexibility and intermobility [16,17,42]. Previous studies on EP systems are often conducted in separate practice settings that show how the EP system plays out in those specific practices, hence lacking the perspective of how the EP system plays out in cross-institutional collaboration, which this study adds. Our findings align with previous studies that highlight physicians’ satisfaction with the EP system in specific practices [14,37] and EP-based work-arounds [16,17]. Still, this study underscores the complexity of cross-institutional and cross-professional collaboration through the EP system, revealing that human coordination is required to enable the EP system to function. Enabling the technology takes place in the medication room and can be considered “invisible work” for others involved in the sociotechnical system. This is in line with another study that emphasized that daily practices and tasks are known to those who perform them but “often remain invisible to managers, health technology vendors and policymakers” [43]. The EP-based work-around for invalid EP is an institutionalized part of the home care nurse work routine but has perhaps remained invisible to other HCPs in the health and care service, health technology vendors, or policy makers. It is in the medication room—where most medication preparation and management occur—that the home care nurse carries out the work-around as the technology delegates new tasks and practices to home care nurses to ensure patient safety. Other studies on systems that exchange patient information across the health and care service find that although the patient information is being shared electronically, the information is perceived as insufficient [21,24]. In addition, telephone and “double communication” are often needed to get nuanced and accurate information, which also applies to our findings when sharing patient information across the health and care service [24]. Our findings can be seen in context with another study, which found a paradox between the home care nurses’ need for information and the struggle to access information when patients transfer from hospital to home due to complicated pathways of exchanging information [22].

The implementation and use of technology in health care services can have unintended consequences [44]. In our findings, the technology delegated new roles, tasks, and responsibilities to the home care nurses, which can be seen as an unintended consequence of the technology that neither the physicians and pharmacists nor technology developers have reflected on or are aware of. In fact, the nurses perform EP-based work-arounds as part of their invisible work to enable the technology. This technology makes the physicians’ workflow easier; however, home care nurses get delegated additional work. An important question is whether this new and invisible work requires attention. This raises questions, such as (1) should this be visualized in the work descriptions of home care nurses? (2) can this be avoided? and (3) should solutions to delegate more work to the EP system be discussed? This could be achieved through alert systems telling GPs to renew their prescriptions. Another important question is whether the regulation of the validity of prescriptions should last longer than 1 year from the issue date. This could lead to a reduction in the number of prescriptions that require renewal. Other studies on systems that exchange patient information across the health and care service found that information is not being shared due to technical hindrances, as the systems “cannot ‘talk to each other’” [23]. On the basis of the findings of our study, one could also imagine that better system integration could be a solution if the pharmacies’ online platforms were integrated with GPs’ EHR systems. Perhaps this could help avoid the need for the home care nurse’s “repeated request loop.”

### Strengths and Limitations

The strength of this study is that it includes multiple perspectives from different units and physicians and nurses. This contributes to understanding how digital systems play out in different contexts across health and care service levels for various HCPs. Another strength of this study is the combination of participant observations and individual interviews.

This study has some limitations. GPs, pharmacists, and pharmacies were not included in this study due to limitations in the scope of the doctoral project that this study is a part of. The first limitation is that we did not include GPs in this study. GPs have an important role in both prescribing and renewing prescriptions for patients. This study emphasizes the critical role GPs play and how the GPs’ responsibilities affect the roles and responsibilities of home care nurses in performing their work in medication management routines. The second limitation is that we did not include the pharmacists or the pharmacies; their perspective on the EP system and their collaboration with the GPs, hospitals, and home care service could strengthen this study. A third limitation is that one researcher conducted the data collection and the empirical inductive coding alone. Including a second or third researcher in the data collection or empirical coding could have been a methodological strength. However, the research team was involved in the analysis through several workshops and meetings where the code groups and the themes were discussed thoroughly between all 4 authors. The fourth limitation is that this study had a limited scope and duration of participant observations, typically associated with ethnographic research. Although the limited scope of this study could be considered a limitation, it was necessary to have a limited scope when conducting participant observations with a limited duration. It sharpened the focus on information flow between and across the health and care services and the use and experience of national eHealth systems. The different units were purposefully sampled as the units had a high degree of patient flow, which increased the possibility of their use and experiences with the national eHealth systems. The fifth limitation of this study is that data collection was conducted during the COVID-19 pandemic. This affected data collection, particularly the participant observations, as the first author strived to keep a distance of 2 m from the HCPs. This affected how and where the observation was carried out in the different units, and sometimes, the first author had to leave a room because there was not enough room for the first author to observe.

### Conclusions

Our study illustrates how a digital system for cross-institutional and cross-professional collaboration might appear well functioning and time saving on one hand but, on the other hand, can be demanding and time consuming. In our study, the physicians found the EP system to ease their workflow and assessed it as well functioning and time saving. In contrast, the home care nurses assessed it as demanding and time consuming and ended up as enablers of a technology that saved time and costs for others. Home care nurses had to develop work-arounds to enable the EP system to work and maintain patient safety.

The questions raised in the Discussion section can be valuable for policy makers by considering extending the validity period of prescriptions beyond 1 year from the issue date. This could reduce the frequency of required renewals and ease the workflow for GPs and home care nurses. On the basis of our findings, system designers could explore solutions to delegate more tasks to the EP system, enhancing efficiency, reducing work-arounds, and reducing the home care nurses’ need to enable the EP system by mediating communication between the GPs and the pharmacies. One potential improvement is implementing automated alert systems that notify GPs when prescriptions need renewal. Such a feature could streamline the renewal process, minimize delays, and improve medication management for HCPs and patients. Integrating the online pharmacy platforms and GPs’ EHR systems could reduce inefficiencies, eliminating the need for home care nurses’ “repeated request loop.”

To understand digital systems intended to be used across a comprehensive national health and care service, it is necessary to conduct research that explores such systems across health and care service levels. Our study showed that well-functioning eHealth systems, such as the Norwegian EP system, require changes in the tasks of human actors, organizational structures, and daily collaboration routines. It changed workflows by removing some tasks from one place in the system, such as paper prescriptions for physicians in our case, but simultaneously required new routines elsewhere, such as in the handling of invalid prescriptions for home care nurses. Thus, the EP system is a part of a sociotechnical system of constant change and ongoing negotiations.

## Data Availability

The datasets generated or analyzed during this study are not publicly available due to ethical restrictions regarding data protection issues and the study-specific consent procedures but are available from the corresponding author on reasonable request.

## Abbreviations

EHR: electronic health record
EP: electronic prescription
GP: general practitioner
HCP: health care professional
SDI: stepwise-deductive induction
